# PHB granules are attached to the nucleoid via PhaM in *Ralstonia eutropha*

**DOI:** 10.1186/1471-2180-12-262

**Published:** 2012-11-16

**Authors:** Andreas Wahl, Nora Schuth, Daniel Pfeiffer, Stephan Nussberger, Dieter Jendrossek

**Affiliations:** 1 Institute of Microbiology, University of Stuttgart, Allmandring 31, Stuttgart, 70550, Germany; 2 Biophysics Department, Institute of Biology, University of Stuttgart, Pfaffenwaldring 57, Stuttgart, 70550, Germany

**Keywords:** Poly(3-hydroxybutyrate) (PHB), Polyhydroxyalkanoate (PHA), PHB granule formation, Storage metabolism, PhaM, Biodegradable polymer

## Abstract

**Background:**

Poly(3-hydroxybutyrate) (PHB) granules are important storage compounds of carbon and energy in many prokaryotes which allow survival of the cells in the absence of suitable carbon sources. Formation and subcellular localization of PHB granules was previously assumed to occur randomly in the cytoplasm of PHB accumulating bacteria. However, contradictionary results on subcellular localization of PHB granules in *Ralstonia eutropha* were published, recently.

**Results:**

Here, we provide evidence by transmission electron microscopy that PHB granules are localized in close contact to the nucleoid region in *R. eutropha* during growth on nutrient broth. Binding of PHB granules to the nucleoid is mediated by PhaM, a PHB granule associated protein with phasin-like properties that is also able to bind to DNA and to phasin PhaP5. Over-expression of PhaM resulted in formation of many small PHB granules that were always attached to the nucleoid region. In contrast, PHB granules of *∆phaM* strains became very large and distribution of granules to daughter cells was impaired. Association of PHB granules to the nucleoid region was prevented by over-expression of PhaP5 and clusters of several PHB granules were mainly localized near the cell poles.

**Conclusion:**

Subcellular localization of PHB granules is controlled in *R. eutropha* and depends on the presence and concentrations of at least two PHB granule associated proteins, PhaM and PhaP5.

## Background

Polyhydroxyalkanoates (PHA) are intracellular storage materials of carbon and energy in many prokaryotes. *Ralstonia eutropha* is the most prominent and best-studied poly(3-hydroxybutyrate (PHB) accumulating bacterium [1-3]. The results of 25 years of research on biosynthesis, maintenance, intracellular degradation (mobilization) and application of PHA meanwhile provide a good picture on the structure and components of PHB granules. PHB granules are composed of an amorphous polymer core that is enclosed by a dense proteinaceous surface layer (for reviews see [4-13]). Polymer and surface layer constitute a multifunctional complex for which the term carbonosomes has been proposed [14]. It is still an open question whether phospholipids [15,16] are part of the PHB granule surface layer in vivo or whether they represent an in vitro preparation artifact that occurs upon cell lysis in course of the isolation process of so-called native PHB granules.

Six types of proteins constitute the proteinaceous PHB surface layer in *R. eutropha*: (i) the PHB synthase (PhaC1) is the key enzyme of PHB synthesis and catalyses the polymerization process of 3-hydroxybutyryl-CoA provided by the central metabolism [9,17,18]. The function of a second - catalytically inactive - PHB synthase, PhaC2 [2] is unknown. However, PhaC2 principally has the capacity to bind to PHB granules in vivo [19]; (ii) phasin proteins (PhaPs), in particular PhaP1, cover most parts of the granule surface and prevent coalescence of granules [20-23]; (iii) PHB depolymerases (PhaZs) are important for reutilization (mobilization) of the polymer during times of starvation [24-28]; (iv) oligomer hydrolases (PhaZb, PhaZc, alternative designation PhaYs) are involved in cleavage of intermediately formed 3-hydroxybutyrate (3HB) oligomers during mobilization [29]; (v) regulatory proteins (PhaRs) regulate expression of selected phasin genes [30,31] and (vi) PhaM represents the prototype of a recently discovered novel type of PHB granule associated protein that has phasin properties but also can bind to DNA [32].

However, despite this considerable amount of knowledge it is still an open question whether PHB granules are formed randomly within the cytoplasm or whether localization of PHB granules is controlled by the bacteria. Several studies using fluorescence microscopy (FM) [33-35] and transmission electron microscopy (TEM) [36,37] were performed in the last decade to address this question. However, the results of these studies were inconsistent. While FM analysis of PHB granule formation in different PHB accumulating species suggested a non random localization of “early” PHB granules in the cell periphery of these species [14,33,34], investigation of PHB granule formation in *R. eutropha* by TEM suggested that PHB granules are formed predominantly in the cell centre near dark stained “mediation elements” [36,37]. Electron cryotomography recently revealed that in *R. eutropha* PHB granules at different stages of PHB accumulation are localized more or less in the cell center whereas a preferential formation of PHB granules in the cell periphery has not been observed [38]. The reason why FM and TEM resulted in apparently contradicting results remained unclear although the studies were performed with the same wild type strain. In recent studies of our laboratory we showed that PhaM can bind to PHB, to phasin PhaP5, to PHB synthase PhaC1 and to DNA [22,32]. Consequently, we decided to reinvestigate PHB granule formation and intracellular localization in *R. eutropha* wild type and in mutants with altered expression of PhaP5 or PhaM. Our results show that PHB granules are localized in close contact to the nucleoid if PhaM is present and are detached from the nucleoid when PhaP5 is over-expressed.

## Results and discussion

### Time course of PHB granule formation in *R. eutropha* HF39 and H16

To study the formation and localization of PHB granules in *R. eutropha* we used *R. eutropha* strains H16 and HF39. Both strains have wild type properties with respect to PHB metabolism and easily form PHB granules during growth on rich media such as NB medium media. Strain HF39 is a spontaneous streptomycin resistant mutant of strain H16 and has often been used in place of strain H16 in conjugation experiments because of simplified counter selection of the donor [39]. In this study, the same results were obtained for both strains with the exception that strain HF39 grew slightly slower and produced in average a lower number of PHB granules than strain H16.

Although *R. eutropha* strains H16 and HF39 intermediately accumulated PHB during growth on NB-medium more than 95% of the cells were free of PHB granules in the stationary growth phase after 24 h. Cells that still had PHB granules after this time period (<5%) often were division-inhibited (cells > 10 μm in length) and many of them were dead as revealed by staining with propidium iodide (images not shown). In conclusion, most living cells of the late stationary growth phase of *R. eutropha* on NB-medium were free of accumulated PHB.

**Figure 1:**
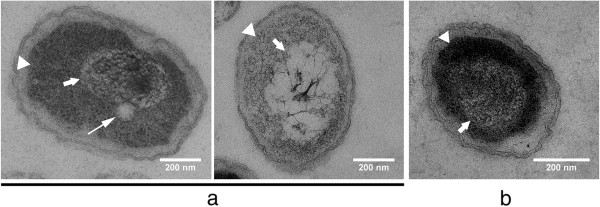
**TEM images of *R. eutropha *H16 (a) and of *R. eutropha *HF39 (b) after 24 h of growth on NB medium (=zero control [t=0 min after transfer to fresh NB-gluconate medium]).** Cells were harvested, fixed and prepared for TEM as described in method section. All thin sections were stained with uranyl-acetate and lead citrate. Arrowheads indicate condensed cytoplasm resulting in an electron-transparent fringe between cytoplasm membrane and cytoplasm. Short arrows indicate the border between cytoplasm and denatured nucleoid. The long arrow in the left cell of (**a**) points to a small globular structure most likely representing an electron-transparent (“white”) remaining, not completely mobilised PHB granule. Note, the PHB granule is in close contact to nucleoid region. Bar represents 0.2 μm.

**Figure 2:**
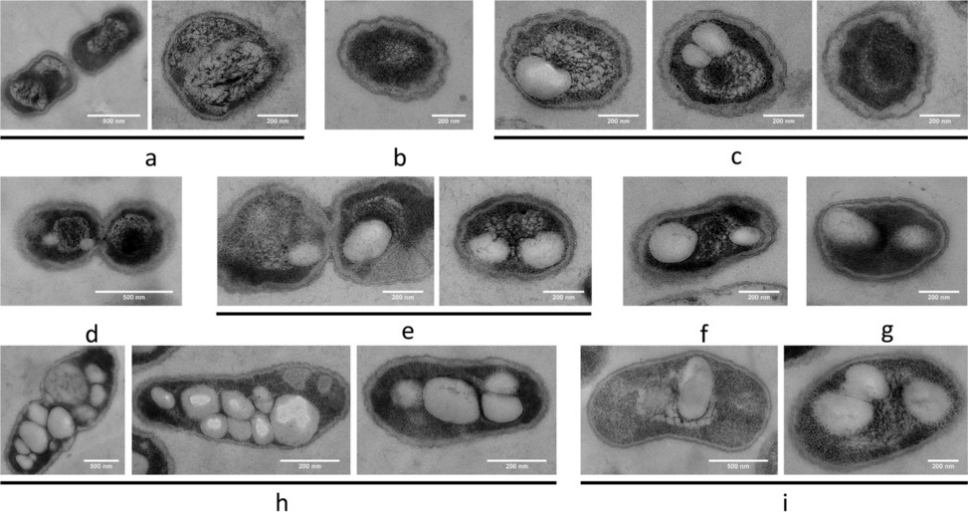
**Time course of PHB granule formation in *R. eutropha *H16 and HF39.** Images of both strains are shown alternately after different times of PHB permissive growth as indicated. All preparations were performed as described in legend to Figure 1. Note, increased number of PHB granules in strain H16 compared to strain HF39 at longer growth times. Strain HF39 [(**a**) 0 min after transfer to fresh NB-gluconate medium; (**d**), 10 min after transfer; (**f**) 40 min and (**i**) 3 hours)]. Strain H16 [(**b**) 0 min after transfer to fresh NB-gluconate medium; (**c**) 10 min; (**e**) 30 min; (**g**) 1 hour and (**h**) 3 hours]. Size of bar as indicated.

**Figure 3:**
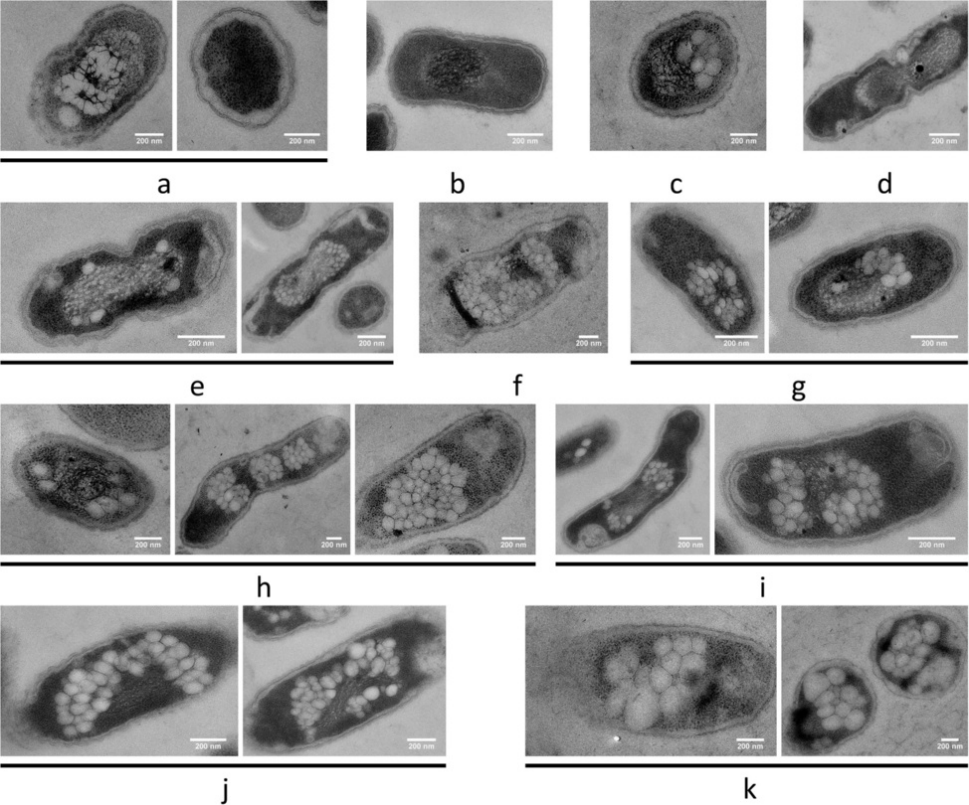
**Time course of PHB granule formation in *R. eutropha *with over-expression of PhaM or eYfp-PhaM.** All preparations were performed as described in legend to Figure. 1. Note, over-expression of PhaM resulted in formation of an increased number of small PHB granules. PHB granules generally were in close contact to nucleoid region. Strain H16 with over-expression of PhaM in (**a**, 0 min; **c**, 10 min; **f**, 40 min; **h**, 60 min; **k**, 240 min). Strain HF 39 (with over-expression of eYfp-PhaM) (**b**, 0 min; **d**, 10 min; **e**, 20 min; **g**, 40 min; **i**, 90 min; **j**, 180 min). Bar 0.2 μm.

**Figure 4:**
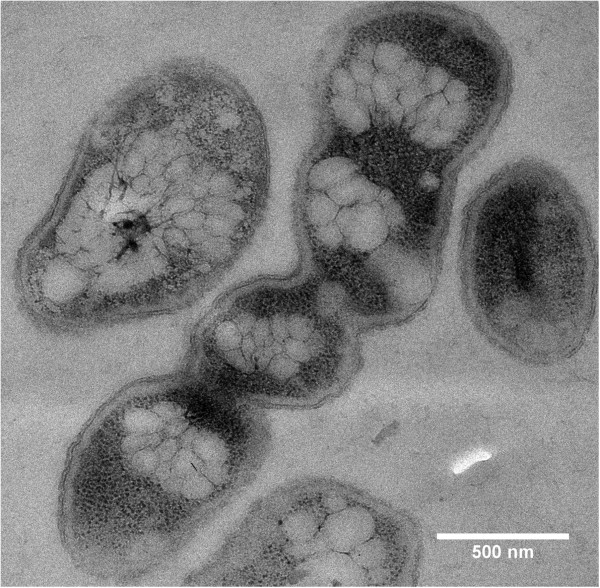
**Individual cell of *R. eutropha *H16 with constitutive over-expression of PhaM after 1 h of PHB permissive conditions.** Three invaginations of the cell wall (= 4 cells) are a visible indication that the last two cell-divisions have not been finished. All preparations were performed as described in legend to Figure 1. Note, presence of four individual, well-separated clusters of PHB granules apparently each bound to the nucleoid regions of the division-inhibited cell. Bar 0.5 μm.

**Figure 5:**
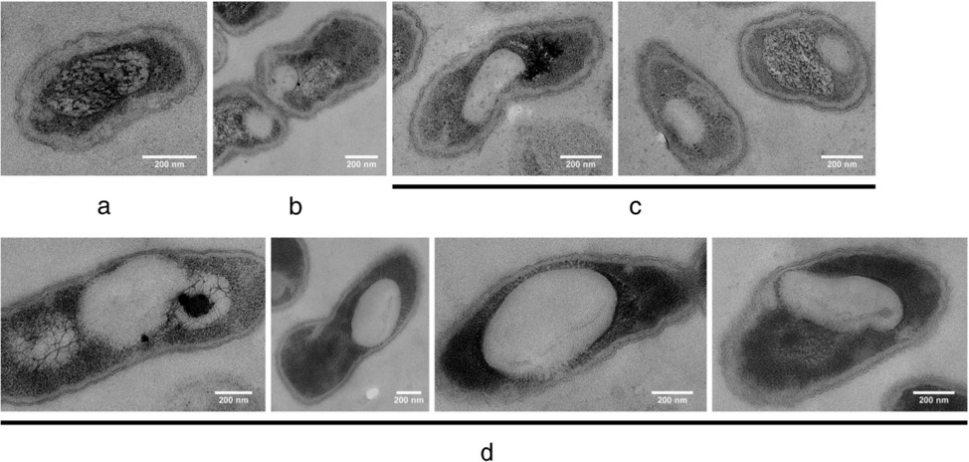
**Time course of PHB granule formation in *R. eutropha *H16 *∆phaM.*** All preparations were performed as described in legend to Figure 1. Note, deletion of *phaM* resulted in formation of decreased number of big PHB granules. Incubation times in NB-gluconate medium for 0 min (**a**), 30 min (**b**), 60 min (**c)** and 180 min in (**d**). Bar 0.2 μm.

**Figure 6:**
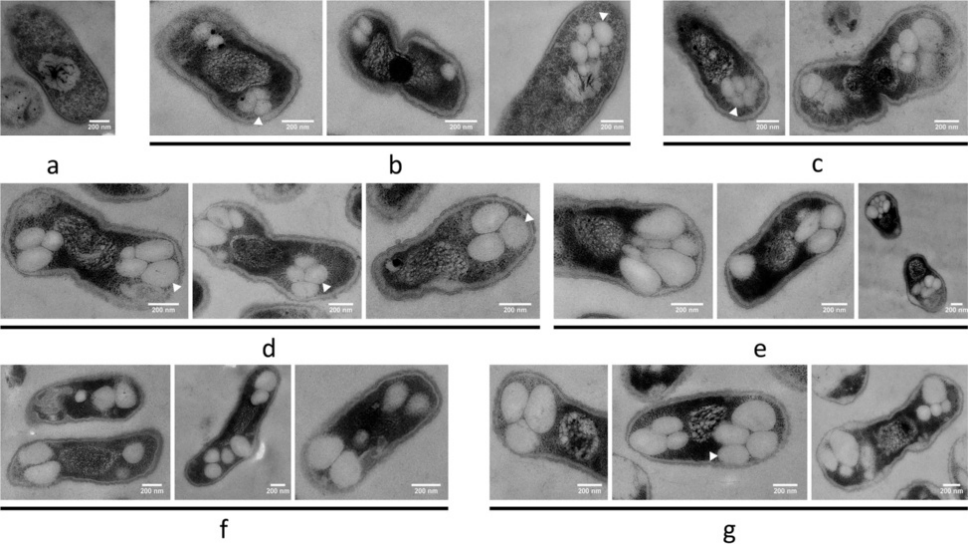
**Time course of PHB granule formation in *R. eutropha *with over-expression of *phaP5.*** All preparations were performed as described in legend to Figure 1. Note, over-expression of *phaP5* resulted in formation of two clusters of 2–5 individual PHB granules. Remarkably, most PHB granules were clearly detached from nucleoid region (arrowheads). Images were prepared from eYfp-PhaP5 over-expressing cells (except for (**f**) in which PhaM was over-expressed in strain H16) to directly compare with cells of Figure 7. No difference was detectable to *R. eutropha* H16 cells with over-expression of PhaP5. Incubation times in NB-gluconate medium for 0 min (**a**), 10 min (**b**), 20 min (**c)**, 40 min (**d**), 90 min (**e** and **f**), 180 min (**g**). Bar 0.2 μm.

To monitor the time course of PHB granule formation we transferred PHB-free stationary *R. eutropha* cells to fresh NB-medium that had been additionally supplemented with 0.2% sodium gluconate. This increased the C to N ratio of the medium and promoted PHB accumulation. Samples were taken at zero time and after 10 min to several hours of growth. Harvested cells were chemically fixed, embedded in a low viscosity acrylic resin and subjected to thin section electron transmission microscopy. PHB granules poorly bind heavy atom stains and therefore have an electron-transparent (“white”) appearance. The results are as shown in Figures 1, 2, 3, 4, 5 and 6.

Figure 1 shows representative images of thin sections of *R. eutropha* H16 at zero time. The cells harvested straight after transfer to fresh medium were rather short rods of about 0.9 μm in length and 0.5 μm in width. Most cells were free of any electron-transparent inclusions. Shortening of cells and consumption of previously accumulated PHB is a typical response of *R. eutropha* to stationary growth phase conditions after growth in NB medium. Despite the fixation procedure of the cells with formaldehyde and glutardialdehyde the cytoplasm often appeared more or less contracted (see arrowheads in Figure 1). This condensing effect of the cytoplasm was stronger in stationary phase cells compared to exponentially growing cells and indicated that the cells become weak in the stationary phase and do not resist the preparation procedure that well. Changing of the fixation conditions, e. g. by increasing the total aldehyde concentration up to 2% and variation of the agar temperature used for embedding of the cells between 46 and 60°C did not prevent formation of preparation artefacts of stationary *R. eutropha* cells such as plasmolysis of fixed cells.

The genomic DNA of the cells denatures during the fixation process and can be identified in stained thin sections by the different degree of staining intensity in comparison to the cytoplasm (see short arrows in Figure 1) [40,41]. In some cells the denatured nucleoids were more intensively stained than in others (e. g. right cell of Figure 1 in comparison to the middle cell). Occasionally (1 to 5% of all cells at zero time), stationary cells revealed small circular structures of about 50–100 nm in diameter with light staining. This structure is likely a remains of small PHB granules (see long arrow in the left cell of Figure 1). PHB is a hydrophobic material and does not bind uranyl acetate or lead citrate that was added to increase the contrast of organic materials in TEM pictures. PHB granules therefore have an electron-transparent appearance. In case of very small PHB granules the diameters of the granules can be smaller than the thickness of a thin-section in transmission electron microscopy. In such cases, or if only a portion of a PHB granule is present within the volume of a thin-section, the appearance of the granules is not a complete “white” but “light grey”. This can be explained by the presence of stained material that was bound to materials of the cytoplasm above or below the granule. In contrast, large PHB granules have a diameter of 300 to 500 nm and are likely to span the complete volume of a thin-section. Large PHB granules therefore appear “white” in TEM images (see large globular structures in Figure 2). Remarkably, the PHB granule visible in Figure 1 (left cell) seems to be attached to the nucleoid region. No difference was observed between strain H16 and strain HF39 at zero time.

When cells were investigated that had been grown under PHB permissive conditions for 10 min to 1 hour many cells harboured one or two PHB granules (Figure 2). All granules were in contact to the nucleoid region. The size of the granules ranged between less than 100 and ≈ 300 nm within the first hour of growth. In cells that harboured two PHB granules the granules mostly were located at opposite sites of the nucleoid region. This observation is in line with previous observations made by fluorescence microscopy (Figure 1C of [19]).

When cells were investigated that had been grown for >1 h permissive for PHB synthesis the number and size of the granules further increased. Strain H16 accumulated in average more granules (up to 12) than strain HF39 (1 to 4). Since the diameter of accumulated PHB granules increased by time the volume of the granules also increased and the association of the granules with the nucleoid became less obvious and could not be differentiated from nucleoid exclusion; however it should be noted that for all cells shown in Figure 2, in which PHB granules and the nucleoid were visible, an association of the granules with the nucleoid is evident. In conclusion, the data suggest that PHB granules are rapidly formed under permissive conditions (within 10 min) and apparently are attached to the nucleoid region. Since PhaM binds to both DNA and PHB we speculated that PhaM is responsible for the association of PHB granules with the nucleoid (see below).

### Time course of formation and subcellular localization of PHB granules in *R. eutropha* that over-express PhaM

PhaM represents a new type of PHB granule associated protein and has multiple functions. It had been identified by its in vivo interaction with PHB synthase PhaC1 in a two-hybrid screening assay [32]. FM analysis revealed that PhaM is not only able to bind to PHB granules but also to the nucleoid region in *R. eutropha*. Moreover, purified PhaM was able to bind to genomic DNA in vitro as indicated in gel mobility shift experiments. To investigate the effect of PhaM on PHB granule formation the *phaM* gene was over-expressed constitutively from the *phaC1* promotor. Figure 3 shows the time course of PHB granule formation in the PhaM-over-expressing transconjugant of *R. eutropha* H16 and HF39. No difference in number, size or localization of PHB granules was found when PhaM-over-expressing cells were compared with eYfp-PhaM over-expressing cells and confirmed that the presence of an eYfp tag did not change subcellular localization of fusion proteins. Most cells were free of PHB granules at zero time and the nucleoid region could be differentiated from the cytoplasm by the different degree of adsorbed staining material similar to wild type cells. PHB granules appeared already after 10–20 min of incubation under PHB permissive conditions. At later time points the number of PHB granules strongly increased up to several dozens. The granules were considerably smaller in diameter (< 100 nm) compared to wild type cells at all stages of growth and the granule size increased only little after longer incubation times at PHB permissive conditions. Remarkably, the granules were not randomly distributed in the cells but were exclusively in contact with or in close neighbourhood to the nucleoid. The PHB granules covered the complete surface of the nucleoid region in some cells. Occasionally, long cells were observed that apparently were inhibited in cell division (Figure 4, 3 h). Such cells harboured several well-separated nucleoid regions each of which was decorated with a high number of attached PHB granules resulting in the impression of “raspberries” within the cells. Free PHB granules, i.e. PHB granules that were not in contact to the nucleoid region were not observed. Apparently, constitutive over-expression of *phaM* resulted in formation of an increased number of small and nucleoid-attached PHB granules.

If PhaM is responsible for the formation of small granules and for the close contact to the nucleoid region, deletion of *phaM* should have a phenotype. In fact, *R. eutropha ∆phaM* cells accumulated only very few (0–2) PHB granules that were significantly larger in diameter than those of the *phaM* over-expressing mutant or of the wild type (Figure 5). Since the diameters of PHB granules of the *∆phaM* strain were considerably larger even at early time points a precise analysis whether or not the granules were attached to the nucleoid region was difficult. In most *∆phaM* cells the PHB granules were still located close to the nucleoid; however, at least in some cells a detachment of PHB granules from the nucleoid region could not be excluded for the wild type or for the *phaM* over-expressing strain. A clear decision whether the absence of PhaM resulted in detachment from the nucleoid can, however, not be made. Since *R. eutropha* expresses at least one other protein with DNA-binding and PHB-binding property (PhaR) [30,31] it might be that PhaR also contributes to association of PHB with DNA. In summary, our data on mutants with altered expression of PhaM clearly show that number, diameter and subcellular localization of PHB granules depends on the presence and concentration of PhaM.

### Time course of formation and localization of PHB granules in *R. eutropha* over-expressing PhaP5

**Figure 7:**
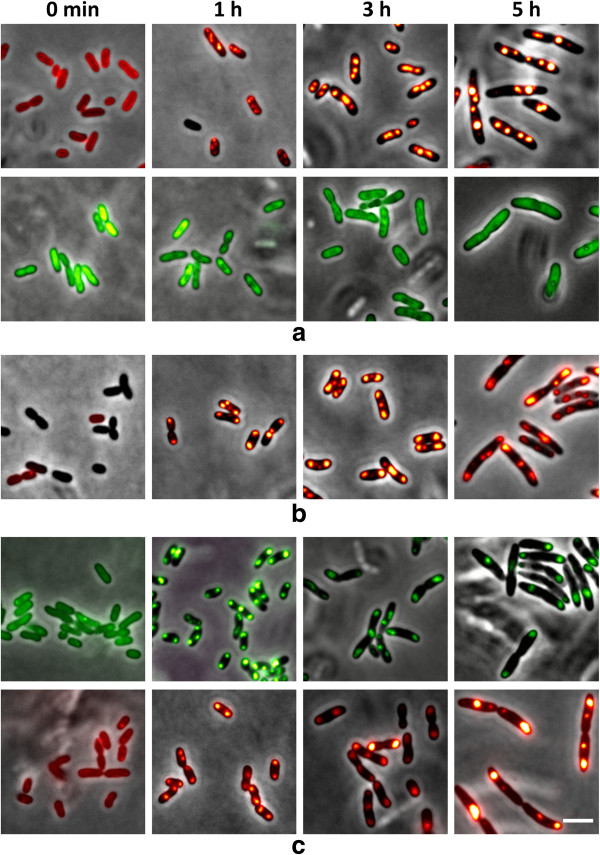
**Fluorescence microscopical (FM) investigation of *R. eutropha *H16 (pBBR1MCS-2-P*phaC*-*eyfp*-c1) with over-expression of eYfp (a); *R. eutropha *H16 (pBBR1MCS-2-P*phaC*-*phaP5*) with over-expression of PhaP5 (b), and *R. eutropha *H16 (pBBR1MCS-2-P*phaC*-eyfp-*phaP5*) with over-expression of eYfp-PhaP5 fusion (c) at various stages of PHB formation.** PHB-free cells from 24 h old seed cultures on NB were transferred to fresh NB medium supplemented with 0.2% gluconate and grown at 30°C. FM-images of samples taken at time points as indicated were generated after staining with Nile red in red channel (top rows) or without staining in green channel (bottom rows) or. Note, individual PHB granules of PhaP5 or eYfp-PhaP5-expressing cells near cell poles or at mid cell were not resolved in FM images as in TEM images (Figure 6). Bar 3 μm.

PhaP5 had previously been identified as a phasin in *R. eutropha* by its in vivo interaction with PhaP2 and other phasins [22]. Remarkably, PhaP5 also interacted with PhaM. To investigate the influence of PhaP5 on PHB granule formation the *phaP5* gene was cloned in a broad host range plasmid (pBBR1MCS-2) under control of the strong and constitutive *phaC1* promotor (P*phaC*), transferred to *R. eutropha* H16 and HF39 via conjugation and investigated for PHB granules formation and localization under PHB permissive conditions (Figure 6). In case of strain HF39 a *eypf-phaP5* fusion was cloned and used to confirm localization of PhaP5 on the PHB granules by fluorescence microscopy. Controls showed that free eYfp is a soluble protein in *R. eutropha* (Figure 7). 

As in the case of PhaM, no difference in number, size and localization of PHB granules was observed for the over-expressed eYfp-PhaP5 fusion in comparison to over-expression of PhaP5 alone. Growth and accumulation of PHB were similar in the recombinant strains as in the wild type. However, when the time-course of PHB granule formation and localization was investigated by TEM-analysis remarkable differences to the wild type were observed for the PhaP5 over-expressing strains (Figure 6): PHB granules were formed in aggregated clusters of in average 2–6 granules in most cells near both cell poles of the rod-shaped cells. These clusters could not be resolved by FM-analysis (Nile red staining) and resulted in the impression of only two (large) PHB granules near the cell poles (Figure 7). The number of individual granules visible in TEM images was increased but the diameter was decreased compared to wild type granules. In most cells, the PHB granule clusters or at least individual PHB granules of a cluster were clearly detached from the nucleoid region (see arrowheads in Figure 6). In conclusion, over-expression of PhaP5 has an impact on number, size and localization of PHB granules and leads to detachment of the granules from the nucleoid. This can be explained by binding of over-expressed PhaP5 to PhaM molecules thus preventing PhaM from binding to DNA and/or to PhaC. Alternatively, a competitive displacement of PhaM molecules from PHB granules surface by over-expressed PhaP5 could be responsible for the phenotype. Number and localization of PHB granules in a *∆phaP5* strain were, however, not significantly changed in comparison to wild type (data not shown).

## Conclusions

Our data clearly show that formation and localization of PHB granules occurs not randomly but is specifically controlled in *R. eutropha*. Other examples of species with non-random localization of PHB granules are *Rhodospirillum rubrum*[33], *Haloquadrata walsbyi*, *Azotobacter vinelandii*, *Beijerinckia indica*[34], *Caryophanon latum*[35] and *Hyphomicrobium facile* (supplementary material of [32]). However, we do not know whether attachment of PHB or PHA granules to the DNA is a general feature of PHB or PHA accumulating bacteria. PHB granules in *R. eutropha* are attached to the nucleoid via PhaM. Our conclusion is supported by previous TEM analysis of others if the “dark-stained mediation elements” are interpreted as denatured chromosomal DNA [36,37]. The reason why the authors of these studies did not observe PHB granules in the cell periphery in thin sections might be explained by differences in growth conditions of the bacteria: they used a mineral salts-based PHB production medium with high carbon but low nitrogen source; first samples were taken after 2.5 h of incubation. At this time the nitrogen source should have been consumed resulting in strong PHB accumulation but also in stop of nucleoid replication. In our experiments, the cells were subjected to high carbon (gluconate) and high nitrogen (nutrient broth) sources resulting in cell growth AND PHB granule formation. Active separation of the replicated chromosomes with bound PHB granules resulted in formation of cells with PHB granules that often localized near the cell poles. Therefore, the results of Tian et al. are not contradictionary to our findings. Moreover, our data are also in agreement with recent biochemical work of the same group in which an association of PHB and PhaM was confirmed [18].

Over-expression of phasin PhaP5 leads to detachment of PHB granules from the nucleoid probably because of competitive binding to PhaM. However, the expression level of PhaP5 in *R. eutropha* wild type is only low as indicated by transcriptome data [42]. An involvement of additional proteins in subcellular localization can not be excluded.

## Methods

### Bacterial strains, plasmids and culture conditions

Bacterial strains and plasmids used in this study are shown in Table 1. All strains of *R. eutropha* were routinely grown in nutrient broth (NB) medium at 30°C. 0.2% (w/v) of sodium-gluconate was added as indicated to promote PHB accumulation. 10 mL nutrient broth (0.8%) in a 100 mL Erlenmeyer flask were inoculated with a single colony of the strain of interest and was incubated for 24 h at 30°C. This seed culture was transferred to 90 ml fresh NB medium (1 L Erlenmeyer flask) and incubated for another 24 h on a rotary shaker. In case of recombinant strains harbouring plasmids 50 μg/mL kanamycin was present in the seed cultures. HF39 cells were grown in the presence of streptomycin (250 μg/mL). The cells intermediately accumulated PHB on NB medium. The bacteria were in the stationary growth phase after 24 h to 30 h of incubation as indicated by shortening of the cells and consumption of previously accumulated PHB. More than 95% of the cells were free of PHB granules as confirmed by fluorescence microscopy after Nile red-staining and by GC analysis of lyophylized cells. Samples of the second seed culture were taken after 24 h to 30 h as zero control for monitoring formation of PHB granules (see below). 10 mL of the second seed culture were used for inoculation of 40 mL of fresh NB-medium (prewarmed to 30°C) and 0.2% sodium gluconate (from 40% sterile stock solution) were added to promote PHB accumulation. This procedure resulted in generation of a quasi-synchronized culture in which all (living) cells immediately started to multiply AND to accumulate PHB. Up to 8 parallel cultures were inoculated and incubated on a rotary shaker at 30C. One culture each was harvested after different periods of incubation (10, 20, 30, 40, 60 min, 3, 4, 5 hours). For this, the culture was transferred to Falcon tubes and immediately cooled on ice. Cells were centrifuged (4°C) and washed with 1 mL of ice-cold PBS (phosphate-buffered saline consisting of 50 mM potassium phosphate and 0.8% NaCl, pH 7.2). Cells were resuspended with 0.8 mL PBS and solutions of formaldehyde (final concentration 0.3 to 1.0%) and glutardialdehyde (0.2 to 1.0%) were added for fixation. Samples were stored on ice overnight.

**Table 1:** Strains and plasmids used in this study

**Strain**	**Relevant characteristic**	**Source or reference**
*Escherichia coli* JM109	Cloning strain	
*E. coli* S17-1	Conjugation strain	[45]
*Ralstonia eutropha* H16	Wild type strain, PHB accumulation	DSMZ 428
*Ralstonia eutropha* HF39	Streptomycin resistant derivate of H16	[22,39]
*R. eutropha* H16 *∆phaP5*	Chromosomal deletion of *phaP5*	[22]
*R. eutropha* H16 *∆phaM*	Chromosomal deletion of *phaM*	[32]
**Plasmid**	**Relevant feature(s)**	**Source or reference**
pBBR1MCS-2	broad host range vector	[46]
pBBR1MCS2- P*phaC-eyfp-c1*	Constitutive eYfp over-expression	[22]
pBBR1MCS-2- P_*phaC*_*-eyfp-phaP5*	Fusion of PhaP5 to C-terminus of eYfp	[22]
pBBR1MCS-2- P_*phaC*_*–eyfp-phaM*	Fusion of eYfp to N-terminus to PhaM	[32]
pBBR1MCS-2- P_*phaC*_*–phaP5*	Constitutive over-expression of PhaP5	this study
pBBR1MCS-2- P_*phaC*_*–phaM*	Constitutive over-expression of PhaM	this study

### Preparation of cells for TEM analysis

Fixed cells were washed three times with 1 mL PBS+10 mM glycine to remove excess of aldehydes. An aliquot of the cells was taken for fluorescence microscopy. The cell pellet of the third washing step was resuspended with PBS in a final volume of 100 μL. Cells were added to an equal volume of a 2% (in PBS) agar solution (prewarmed to 50°C in a 2 mL Eppendorf tube using prewarmed pipette tips), mixed and centrifuged for ≈ 10 s at room temperature to obtain a high cell concentration at the bottom of the agar. The agar was cooled on ice. The agar block containing fixed *R. eutropha* cells was removed from the Eppendorf cups using a steam of nitrogen gas applied with a capillare to the bottom of the Eppendorf tube and was cut into more or less cube-shaped pieces (≈ 1 mm^3^). The cells were dehydrated by incubation of the agar cubes in a series of subsequent dehydration steps using: 15% methanol on ice for 15 min, 30% ethanol for 30 min on ice, and subsequent 30 min incubation steps at - 20°C using 50%, 70%, 96% and 100% (twice) ethanol. Subsequently, the dehydrated cubes were transferred to a solution consisting of ethanol and LR white resin (3:1) and incubated at room temperature for 2 h before the solution was exchanged against pure LR white and incubated at 4°C for at least 2 h (or overnight). Several cubes were then transferred to gelatine capsules, filled with LR white and polymerized at 50°C (or 60°C) for 30 h (or 24 h). The solidified samples were stored in the dark at room temperature until use. Thin sections were prepared using a Leica Ultracut microtome and glass or diamond knifes. Slices of appropriate thickness were transferred to copper grids and stained with uranyl acetate (2%) and lead citrate according to Reynolds [43]. EM images of thin sections were recorded using a Tecnai G2 Sphera electron transmission microscope (FEI) equipped with a large area TemCam F224HD CCD camera (TVIPS). The microscope was operated at 120 kV.

### Over-expression of PhaM and PhaP5

The *phaM* and *phaP5* genes were cloned under control of the (in *R. eutropha*) constitutively expressed *phaC* promoter in pBBR1MCS2-P*phaC* (Table 1). The primer sequences (PhaP5_f_NdeI GGGAATTCCATATGGCCACGCCTCCCAATCC, PhaP5_r_BamHI CGGGATCCCTAGCCCTTGGATTTCGGCTTG and PhaM_f_NdeI GGGAATTCCATATGTTCGGACAGATTCCCGATTTC, PhaM_r_BamHI CGGGATCCTCAGGCTGCGCTGCTG) were used for amplification of *phaP5* and *phaM*. The respective PCR products were ligated into pBBR1MCS2-P*phaC via NdeI* &*BamHI* sites and cloned in *E. coli* JM109*.* Integration and DNA sequence of cloned genes were verified by determination of the DNA sequence. Plasmids were conjugatively transferred from. *E. coli* S17-1 harbouring the plasmid of interest were conjugatively transferred to *R. eutropha* H16 or strain HF39 by selection on mineral salts medium supplemented with 0.5% fructose and 350 μg ml^-1^ kanamycin as described previously [22,32]. The respective strains were grown on NB medium supplemented with 0.2% gluconate as described above. Strains with constitutively expressed fusions of PhaM or PhaP5 with eYfp were expressed in an analogue way.

### Other methods

Molecular biological experiments were performed by standard methods [44]. Fluorescence microscopical analysis of *R. eutropha* cells harbouring fusion proteins with eYfp in the absence or presence of Nile red was conducted as described previously [34]. Construction of chromosomal deletions of *phaP5* and of *phaM* in *R. eutropha* strains has been described elsewhere [22,32] using a *sacB*-based system for selection of double cross-over events. In all cases the mutations were verified by PCR-amplification of the mutated gene locus and by determination of the amplified DNA sequence. Only clones with correct DNA sequence were used.

## Competing interests

The authors declare that they have no competing interests.

## Authors’ contributions

NS and AW carried out most TEM experiments. DP constructed the recombinant strains and performed FM experiments. DJ designed the experiments and wrote the manuscript. SN introduced the coauthors to TEM technology. All authors read and approved the manuscript.
